# Advanced Photocatalytic Materials for Environmental and Energy Applications

**DOI:** 10.3390/ma16227197

**Published:** 2023-11-17

**Authors:** Tongming Su, Xingwang Zhu

**Affiliations:** 1 School of Chemistry and Chemical Engineering, Guangxi University, Nanning 530004, China; 2 School of Environmental Science and Engineering, Institute of Technology for Carbon Neutralization, Yangzhou University, Yangzhou 225009, China

With the development of modern society, environmental pollution and energy shortage have become the focus of worldwide attention. A majority of the global energy supplies are generated from fossil fuel, which gives rise to environmental pollution and climate change. As an inexhaustible clean energy source, solar energy has been widely researched and utilized for decades. Photocatalytic technology, which can directly convert solar energy into high value-added chemical energy and chemical materials or degrade a wide range of organic pollutants into easily degradable intermediates or less toxic small molecular substances, is regarded as one of the most crucial ways to solve the global energy shortage and environmental pollution problem.

This Special Issue includes two reviews and nine original research articles prepared by scientists from different countries, and these works focus on advanced photocatalytic materials for the treatment of indoor air, photocatalytic bacterial inactivation, photocatalytic hydrogen evolution, photocatalytic oxygen evolution, photocatalytic CO_2_ reduction, photocatalytic hazardous pollutant removal, photothermal decomposition of pollutants, and photoelectrochemical water splitting. This Special Issue provided a platform for scientists to present their original research on “Advanced Photocatalytic Materials for Environmental and Energy Applications”. The following is a brief summary of each of the papers that we have had the honor of editing to highlight recent advances in the utilization of photocatalytic materials for environmental and energy applications.

Indoor air quality has become a significant public health concern, and photocatalytic technology has been widely studied for environmental remediation, especially for air treatment. Assadi et al. [1] summarized the recent developments on the TiO_2_-based materials for indoor air treatment and bacterial inactivation. In addition, several strategies, such as doping, heterojunction techniques, and combined catalysts, for enhancing the photocatalytic activity of TiO_2_-based catalysts was discussed. Moreover, the catalysts used to remove volatile organic compounds and microorganisms was reviewed. Finally, the reaction mechanism of pollutant elimination and microorganism inactivation using photocatalytic technology was also summarized.

The greenhouse effect exerts a great influence on mankind, and the photocatalytic reduction of CO_2_ into valuable products such as CO and CH_4_ is considered a promising technology for alleviating the greenhouse effect. Qin et al. [2] synthesized the phosphorus-doped hollow tubular g-C_3_N_4_ (x-P-HCN) and used it for photocatalytic CO_2_ reduction. They found that the phosphorus-doped g-C_3_N_4_ can effectively activate the CO_2_ adsorbed on the surface of photocatalysts, which greatly enhanced the CO production rate of photocatalytic CO_2_ reduction. Among the x-P-HCN samples, the 1.0-P-HCN sample exhibited the largest CO production rate of 9.00 µmol·g^−1^·h^−1^, which was 10.22 times higher than that of bulk g-C_3_N_4_. This work provided an in-depth research perspective for accelerating the photocatalytic CO_2_ reduction rate of g-C_3_N_4_.

Outdoor air pollutants originating from familiar anthropogenic sources, including industry, transportation, heating, and agriculture, exhibit adverse effects on health, the environment, the planet, and climate. In recent years, the dielectric barrier discharge (DBD) reactors have been widely used for hazardous pollutant removal. Khezami et al. [3] investigated the pilot-scale combination of non-thermal plasma and photocatalysis for removing toluene and dimethyl sulfur and revealed the effects of plasma energy and initial pollutant concentration on the performance and by-product formation in both pure compounds and mixtures. They found that coupling DBD plasma with a TiO_2_ catalyst can achieve a synergetic effect, which resulted in improved toluene and dimethyl sulfur removal. In addition, the ozone can be reduced, and CO_2_ selectivity was enhanced by combining plasma with photocatalysis.

Phase engineering is an effective strategy for tuning the electronic states and catalytic performance of photocatalysts, such as the light absorption range, charge separation efficiency, and surface redox reactivity. Yi et al. [4] provided a critical insight on the classification and diversified applications of phase-engineered photocatalysts. In this review, the classification of phase engineering for photocatalysis was summarized, and the applications of phase-engineered photocatalysts in hydrogen evolution, oxygen evolution, CO_2_ reduction, and organic pollutant removal was reviewed. In addition, the synthesis and characterization methodologies for unique phase structures and the correlation between phase structure and photocatalytic performance was introduced. Finally, the current opportunities and challenges of phase engineering for photocatalysis was also discussed.

Photothermal catalysis, which combines photochemical and thermochemical contributions of sunlight, has attracted a great amount of attention in the field of pollutant decomposition. Tryba et al. [5] prepared the Ni-TiO_2_ photocatalyst with nickel foam as the support and used it for the photothermal decomposition of acetaldehyde. They found that with the nickel foam as the support, the photocatalytic acetaldehyde decomposition performance over TiO_2_ can be enhanced from 31% to 52% under room temperature, and from 40% to 85% at 100 °C. In addition, the mineralization of acetaldehyde into CO_2_ doubled in the presence of nickel foam. This enhanced performance was attributed to the synergistic effect between nickel foam and TiO_2_, which can enhance the separation of free carriers and provide more space for the interaction between the reactant and the photocatalyst.

In recent years, the photoelectrochemical process was considered as an effective way to generated clean, green hydrogen (H_2_) directly from water using solar energy. Arifin et al. [6] successfully decorated Mo_2_C on TiO_2_ nanotube arrays (NTs) and used them for photoelectrochemical water splitting. They found that the photocurrent density was greatly increased from 0.21 mA cm^−2^ to 1.4 mA cm^−2^ when TiO_2_ NTs were decorated with Mo_2_C. This enhanced photocurrent density may have been due to the fact that the Mo_2_C cocatalyst greatly improved the photocatalytic characteristics of the TiO_2_ NTs. This work provides an effective method for developing the photoelectrochemical water splitting devices.

As already well known, process optimization is a feasible strategy to enhance the efficiency of polycrystalline silicon solar cells in the photovoltaic industry. To improve the efficiency of polycrystalline silicon solar cells, Wang et al. [7] developed a “low-high-low” temperature step of the POCl_3_ diffusion process. Compared with the online low-temperature diffusion process, the open-circuit voltage and fill factor of solar cells increased up to 1 mV and 0.30%, respectively, in the optimization diffusion process. In addition, the efficiency of solar cells and the power of PV cells were also increased by 0.1% and 1 W, respectively. These enhancements can be ascribed to the low surface concentration of P doping and the strong impurity absorption effect of Si wafers obtained from the low-high-low temperature diffusion process.

Harnessing solar energy has been revealed to be a green strategy for solving the environmental pollution and energy crises. To make full use of solar energy, visible light-responsive photocatalysts have attracted a wide range of attention. Hu et al. [8] synthesized a novel carbon quantum dots (CQDs)-modified PbBiO_2_I photocatalyst and used it for the photocatalytic degradation of organic contaminants under visible/near-infrared light irradiation. After PbBiO_2_I was modified with CQDs, the photocatalytic performance for the degradation of rhodamine B and ciprofloxacin was significantly enhanced under visible/near-infrared light irradiation. The enhanced photocatalytic performance can be ascribed to the following reasons: CQDs can absorb light in the near-infrared region, and the modification of CQDs enhanced the separation of photogenerated electrons and holes and increased the contact between the catalyst and the organic molecules. This work expanded the application of CQDs in the fields of photocatalysis and solar energy conversion.

Environmental issues, such as air pollution and water pollution, directly affect our lives. The photocatalytic degradation of organic pollutants in water is an effective method to deal with water pollution. Dai et al. [9] prepared a novel Co_3_O_4_/g-C_3_N_4_ composite photocatalyst and used it for the photocatalytic degradation of rhodamine B in water. They found that the type II heterojunction was formed between the Co_3_O_4_ and g-C_3_N_4_, and that the type II heterojunction significantly enhanced the transfer and separation of photogenerated electrons and holes in the Co_3_O_4_/g-C_3_N_4_ photocatalyst. Therefore, the photocatalytic performance for the degradation of rhodamine B over optimized 5% Co_3_O_4_/g-C_3_N_4_ photocatalyst was 5.8 times higher than that of g-C_3_N_4_. In addition, they found that the main active species for the degradation of rhodamine B are •O^2−^ and h^+^.

In order to promote the photocatalytic degradation of methylene blue in water, Tao et al. [10] successfully synthesized the MoS_2_/SnS_2_ composite photocatalysts via a facile hydrothermal method. They found that the SnS_2_ nanosheets were grown on MoS_2_ nanoparticles under a smaller size, and that a heterojunction was formed between MoS_2_ and SnS_2_. The optimal MoS_2_/SnS_2_ composite exhibited a methylene blue degradation efficiency of 83.0%, which was 8.3 times and 16.6 times higher than that of MoS_2_ and SnS_2_, respectively. The enhanced photocatalytic performance of the MoS_2_/SnS_2_ composite can be attributed to the improved visible light absorption, more active sites at the exposed edges of MoS_2_, and the enhanced separation of photogenerated electrons and holes.

Mizael et al. [11] synthesized ZnO nanoparticles with *Prosopis laevigata* extract as a stabilizing agent, the prepared ZnO nanoparticles were used for the photocatalytic degradation of methylene blue, and the degradation kinetics were investigated using the LHHW model. They found that the LHHW model sufficiently fits the experimental data, and that the size of the ZnO nanoparticles can be controlled using different concentrations of *Prosopis laevigata* as a reducing and stabilizing agent. In addition, the number of active sites increased with the decreasing nanoparticle size, which was demonstrated using the LHHW model. This work indicated that the extract of *Prosopis laevigata* can be used as a reducing and stabilizing agent to control the size and the photocatalytic performance of ZnO nanoparticles.
